# Age-related trajectories of blood lipids and lipoproteins by sex, region, and waist circumference changes in Korea: a longitudinal cohort study

**DOI:** 10.4178/epih.e2025066

**Published:** 2025-12-09

**Authors:** Mi Kyoung Son, Nam-Kyoo Lim, Joong-Yeon Lim, Hyun-Young Park

**Affiliations:** 1 Division of Population Health Research, Department of Precision Medicine, National Institute of Health, Chungju, Korea; 2 National Institute of Health, Chungju, Korea

**Keywords:** Lipids, Lipoproteins, Waist circumference, Longitudinal studies, Sex

## Abstract

**OBJECTIVES:**

This study aimed to evaluate the longitudinal trajectories of lipid and lipoprotein levels with aging according to sex and changes in waist circumference (ΔWC) from midlife to late life.

**METHODS:**

We included 4,345 male and 4,804 female participants aged 40-69 years at baseline from the Korean Genome and Epidemiology Study (2001-2018). The annual ΔWC was estimated using linear regression. Marginal models were fitted using mixed-effects regression.

**RESULTS:**

The trajectories of total cholesterol (TC), non-high-density lipoprotein cholesterol (non-HDL-C), and low-density lipoprotein cholesterol (LDL-C) levels displayed an increasing trend until the 60s age range in females (approximately 10-15 mg/dL) and the late 40s in males (approximately 3-5 mg/dL), with a subsequent decline. In females, HDL-C levels increased until the early 50s, declined thereafter, and rose again from the 70s onward, with a more pronounced rise in urban than in rural areas, while remaining relatively stable in males. Triglyceride (TG) levels decreased with advancing age in males, whereas in females they increased up to the age of 70 years, followed by a decrease. Females exhibited greater increases in TC, non-HDL-C, HDL-C, LDL-C, and TG across all ages compared with males. Both males and females with a decrease in waist circumference (WC) during follow-up showed improvements in lipid and lipoprotein profiles relative to those with stable or increased WC.

**CONCLUSIONS:**

Trends in lipid and lipoprotein levels vary according to age and sex, and a decrease in WC significantly improves lipid and lipoprotein profiles.

## GRAPHICAL ABSTRACT

[Fig f4-epih-47-e2025066]

## Key Message

• In a prospective cohort analysis of Korean adults aged 40 years and older, lipid and lipoprotein trajectories varied by age, sex, and region, with total cholesterol, low-density llipoprotein cholesterol, and non-high-density lipoprotein cholesterol levels generally showing less favorable patterns in urban than rural areas.

• Females exhibited greater increases across all five lipid and lipoprotein measures than males, while high-density cholesterol levels in females declined around the menopausal transition and increased again in later life.

• A sustained decrease in waist circumference was associated with improvements in lipid and lipoprotein profiles, highlighting the importance of age- and sex-specific approaches to abdominal obesity management.

## INTRODUCTION

Dyslipidemia, characterized by an unfavorable blood lipid profile, is a crucial modifiable risk factor for cardiovascular disease (CVD) [1]. High levels of total cholesterol (TC), low-density lipoprotein cholesterol (LDL-C), and triglycerides (TG) and lower levels of high-density lipoprotein cholesterol (HDL-C) are major lipid risk factors for coronary heart disease [2-5]. Evidence of age-related changes in lipid and lipoprotein levels is primarily based on comparisons of average levels in sequential cross-sectional surveys, with few studies investigating trends over time within the same population [6-9].

According to the results of these studies, age-related changes in lipid and lipoprotein levels are mostly unfavorable: TC, LDL-C, and TG levels decrease after middle age, while the change in HDL-C levels is inconsistent—decreased, unchanged, or increased. Clinically significant sex differences in cardiovascular risk factors have been observed, necessitating increased awareness [10]. With increasing age, serum cholesterol levels substantially increase in females [11]. The reduction in TC levels is greater in males than in females [10], and the prevalence of obesity is higher in females than in males [12].

A multinational South Asian cohort study revealed significant population attributable fractions for CVD associated with high non-HDL-C levels (11.1%) and abdominal obesity (6.9%) [13]. A reduction in waist circumference (WC), reflecting the redistribution of abdominal fat, is hypothesized to positively affect the cardiovascular risk profile [14]. However, limited evidence is available regarding the effects of changes in WC on age-related trajectories of lipid and lipoprotein levels.

Therefore, the present study aimed to identify the sex-specific age-related trajectories of TC, LDL-C, HDL-C, TG, and non-HDL-C levels and to determine whether WC modifies these trajectories, given its public health and clinical implications.

## MATERIALS AND METHODS

### Study population

Between 2001 and 2002, 10,030 Korean male and female participants aged 40-69 years participated in the first phase of the Ansan–Ansung (urban–rural area) cohort of the Korean Genome and Epidemiology Study (KoGES), an ongoing prospective cohort study conducted by the Korea National Institute of Health [15]. The participants have been followed up every 2 years since then. At each visit, all variables used in this study, including medical history, blood tests, clinical examinations, and lifestyle factors, were assessed. Our analyses included participants who underwent at least 2 examinations (2-9 visits; mean number of visits, 7.7) between 2001 and 2018. Participants with fewer than 2 valid measurements for TC, HDL-C, TG, or WC during the study period were excluded. LDL-C was estimated using the Friedewald formula, which is not applicable when TG exceeds 400 mg/dL; therefore, individuals without at least 2 valid LDL-C values during the study period were also excluded. Ultimately, 9,149 individuals were included in the analysis.

### Blood lipid levels

TC, HDL-C, LDL-C, and TG levels were measured using an Advia 1650 clinical chemistry analyzer (Siemens, New York, NY, USA) with dedicated reagents. LDL-C was estimated using the Friedewald formula (LDL-C=TC−HDL-C−TG/5) when TG levels were ≤400 mg/dL. Non-HDL-C levels were calculated by subtracting HDL-C from TC.

### Assessment of waist circumference change

WC was measured 3 times at each visit by trained research staff using a non-elastic tape at the midpoint between the lowest rib and the iliac crest, with the participant in the standing position. The average of the 3 measurements was used in the analysis. To characterize long-term WC trends, person-specific linear regression slopes (cm/yr) were estimated from all available repeated WC measurements during the follow-up period and used as the annual WC change (ΔWC) for each individual. Participants were categorized into 3 groups based on the quartile distribution of ΔWC: decrease (below the first quartile, <0 cm), stable (between the first and third quartiles, 0-0.49 cm), and increase (above the third quartile, >0.50 cm). For example, a slope of 0.50 cm/yr represents a mean annual increase of 0.50 cm in WC. Over a mean follow-up of 13.1 years, this would correspond to an approximate cumulative increase of 6.55 cm in WC, assuming a constant linear rate of change. Abdominal obesity was defined as a WC >85 cm in females and >90 cm in males [16].

### Assessment of clinical and lifestyle-related variables

Information regarding the presence of CVDs, including myocardial infarction, coronary artery disease, congestive heart failure, and stroke/transient ischemic attack, was obtained using a questionnaire at each visit. Trained interviewers administered the questionnaires according to a standardized protocol. Leisure-time physical activity (LTPA), including aerobics, jogging, swimming, tennis, golf, bowling, fitness club exercise, walking, and climbing, was assessed using a questionnaire to quantify activities in the leisure-time domain. LTPA was categorized as no physical activity (inactive) or >0 min/wk (active). Information on the presence of diabetes mellitus, defined as a fasting serum glucose level ≥126 mg/dL, a 2-hour post-load glucose level ≥200 mg/dL after a 75-g oral glucose tolerance test, a glycated hemoglobin level ≥6.5%, or current antidiabetic treatment, was also collected. Systolic blood pressure (SBP) and diastolic blood pressure (DBP) were recorded as the average of 3 physician-obtained measurements during the examination. Hypertension was defined as SBP/DBP ≥140/90 mmHg or current use of antihypertensive medications.

### Statistical analysis

The characteristics of the study population at the first and last attended examinations are described as frequencies (percentages) for categorical variables and means±standard deviations for continuous variables.

Mixed-effects regression models were used to separately estimate lipid and lipoprotein trajectories in male and female participants, with age used as the common timescale for all analyses. Repeated lipid and lipoprotein measurements were used to fit mixed-effects linear regression models, with each lipid or lipoprotein level as the outcome, participant identification as a random intercept, and age as a fixed effect modeled with both linear and quadratic terms to allow for non-linear relationships. Mean values were estimated for each lipid and lipoprotein measure over the age range of 40-86 years in males and females. To account for sex-specific physiology, lipid and lipoprotein changes from baseline levels and the differences in these changes between females and males were subsequently calculated. To investigate cohort effects on the trajectories of lipid and lipoprotein levels, participants were stratified according to their baseline age group (40s, 50s, and 60s), and trajectories were estimated within each group. For graphical presentation and to assess the functional form of the age effect, smoothed trajectories were additionally plotted using restricted cubic splines with 4 knots. The influence of ΔWC was also examined. Lipid and lipoprotein levels and covariates, including WC, that were not recorded at a study visit were treated as missing. The mixed-effects models incorporated all available repeated measurements under the assumption that data were missing at random, and no imputation was performed. Mixed-effects regression models were further adjusted for time-varying smoking status, alcohol consumption, LTPA, hypertension, diabetes mellitus, CVD, lipid-lowering medication, WC, and examination date, as well as baseline area and education level.

All statistical tests were two-sided, and p-values of less than 0.05 were considered to indicate statistical significance. All analyses were performed using SAS version 9.4 (SAS Institute, Cary, NC, USA).

### Ethics statement

The study protocol was approved by the Institutional Review Board of the Korea Centers for Disease Control and Prevention (IRB No. 2017-05-04-4C-A). All research procedures were performed in accordance with relevant guidelines and regulations.

## RESULTS

The characteristics at baseline and at the final examination according to sex are presented in Table 1. Of the 9,149 participants, approximately half (47.5%) were male. The average number of visits was 7.7, and the average follow-up duration was 13.1 years (Supplementary Material 1). Among the study population, 67.3% were followed until the final survey wave in 2017-2018 (Supplementary Material 2).

**Figure 1.**
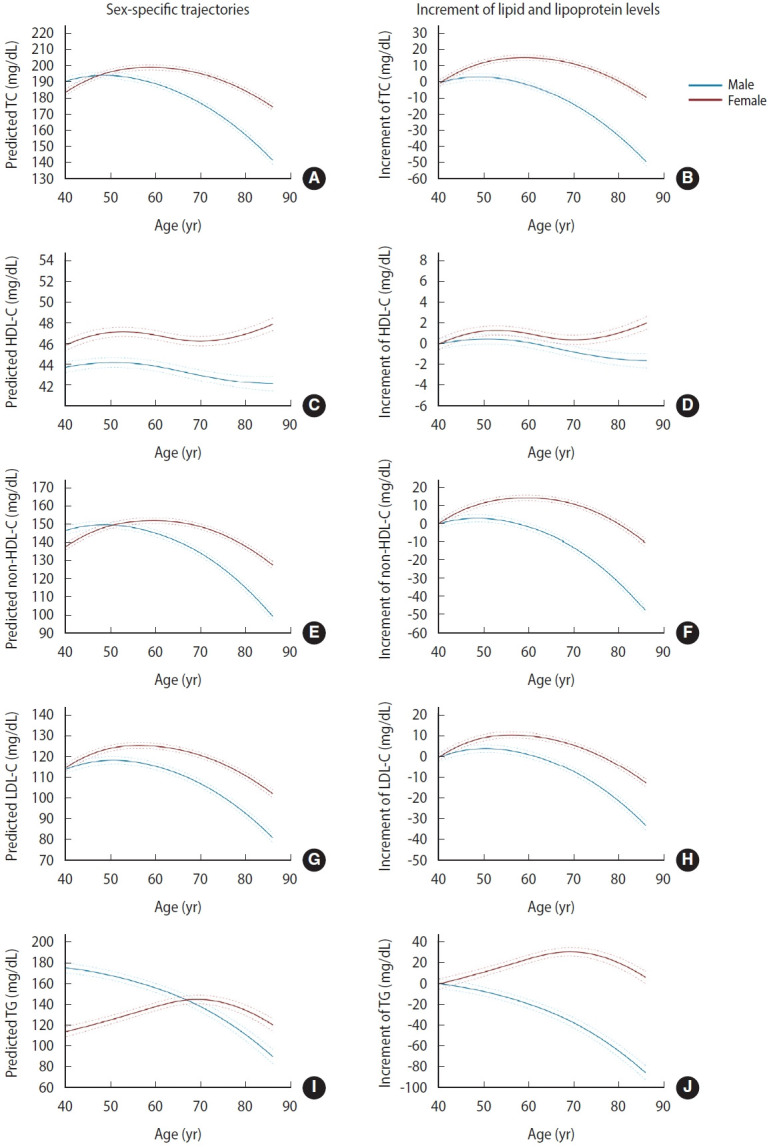
Sex-specific trajectories of blood lipid and lipoprotein levels with age. Predicted mean trajectories and corresponding changes from baseline in TC (A, B), HDL-C (C, D), non–HDL-C (E, F), LDL-C (G, H), and TG (I, J) are shown for males and females. The blue and red solid lines represent the trajectories of males and females, respectively, and dashed lines represent 95% confidence intervals for each sex category. Adjusted for time-varying smoking status, alcohol consumption, leisure time physical activity, hypertension, diabetes mellitus, cardiovascular disease, lipid-lowering medications, examination date, and waist circumference, as well as baseline area and education level. Predicted trajectories were obtained from mixed-effects spline models, with covariates held constant at their mean values. TC, total cholesterol; HDL-C, high-density lipoprotein cholesterol; LDL-C, low-density lipoprotein cholesterol; TG, triglyceride.

**Figure 2.**
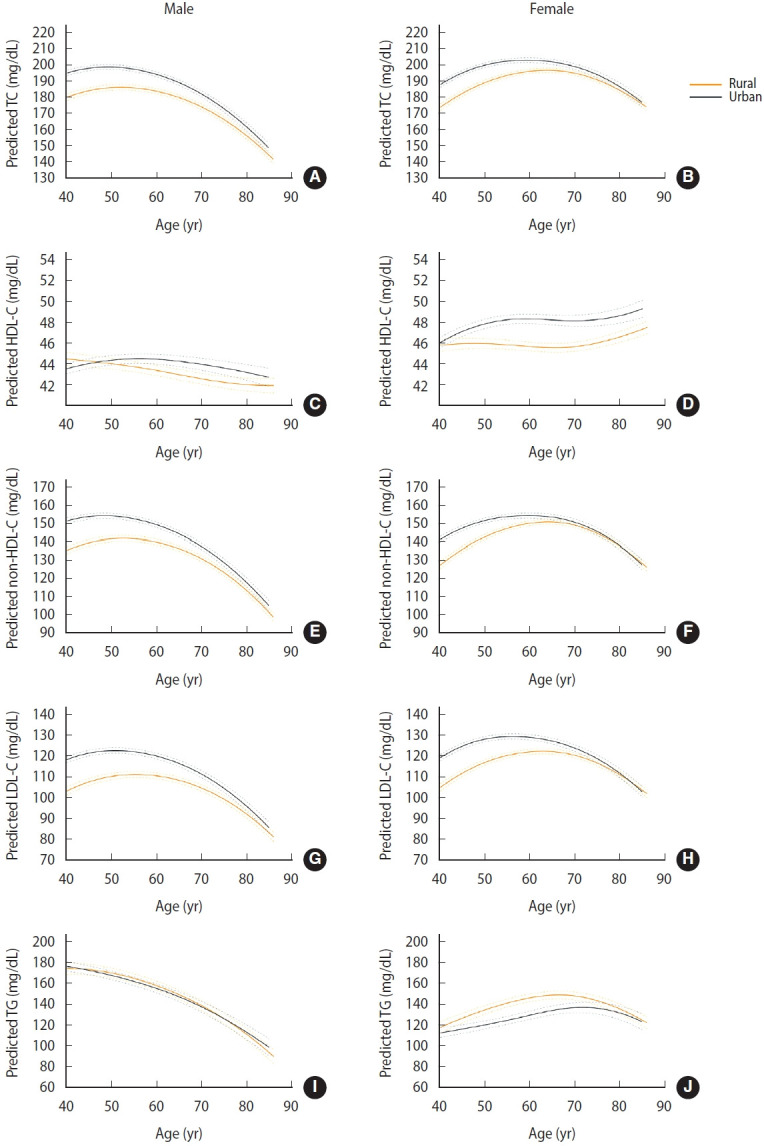
Estimated trajectories of blood lipid and lipoprotein levels with age according to sex and area. Predicted mean trajectories of TC are shown in males (A) and females (B), HDL-C in males (C) and females (D), non-HDL-C in males (E) and females (F), LDL-C in males (G) and females (H), and TG in males (I) and females (J). The yellow and gray solid lines represent the trajectories for rural and urban areas, respectively, dashed lines represent 95% confidence intervals for each area. Adjusted for time-varying smoking status, alcohol consumption, leisure time physical activity, hypertension, diabetes mellitus, cardiovascular disease, lipid-lowering medications, examination date, and waist circumference, as well as baseline area and education level. Predicted trajectories represent covariate-adjusted marginal means estimated from mixed-effects spline models, reflecting an average over the empirical distribution of covariates: continuous covariates were held constant at their sample means, and categorical covariates were weighted by their observed proportions. TC, total cholesterol; HDL-C, high-density lipoprotein cholesterol; LDL-C, low-density lipoprotein cholesterol; TG, triglyceride.

Lipid and lipoprotein levels displayed significant associations with sex and a sex–age interaction, as well as persistently inverse associations with age squared (all p<0.05) in both males and females, indicating that sex differences varied across age and that lipid and lipoprotein levels followed inverted U-shaped (quadratic) trajectories with age (Table 2). In females, TC, non-HDL-C, and LDL-C levels exhibited an increasing trend until the mid-60s, with a subsequent decline (Figure 1A). In males, these markers increased until the late 40s and then decreased. In females, HDL-C levels increased until the early 50s, declined thereafter, and rose again from the 70s onward; however, the magnitude of these changes was modest. In males, HDL-C levels remained relatively stable across age. TG levels decreased with advancing age in males, whereas in females, they increased up to the age of 70 years and then decreased. The observed patterns remained consistent even after excluding participants who were on lipid-lowering medications, confirming the robustness of the findings (Supplementary Material 3). Females exhibited greater increases in all 5 lipid and lipoprotein measures across all ages compared with males (Figure 1B). Females had lower TC and non-HDL-C levels than males until the late 40s and early 50s, respectively, with the pattern reversing thereafter. Lipid and lipoprotein levels were generally higher in urban than in rural areas, whereas TG levels showed the opposite trend (Figure 2). For HDL-C, males in urban areas showed a slight increase until the 60s, followed by a modest decline, whereas those in rural areas exhibited a continuous slight decrease (Figure 2A). In females, those in urban areas showed an increase until the late 50s, stability thereafter, and a renewed rise from the mid-70s, while those in rural areas displayed little change until the late 60s and then increased thereafter (Figure 2B).

**Figure 3.**
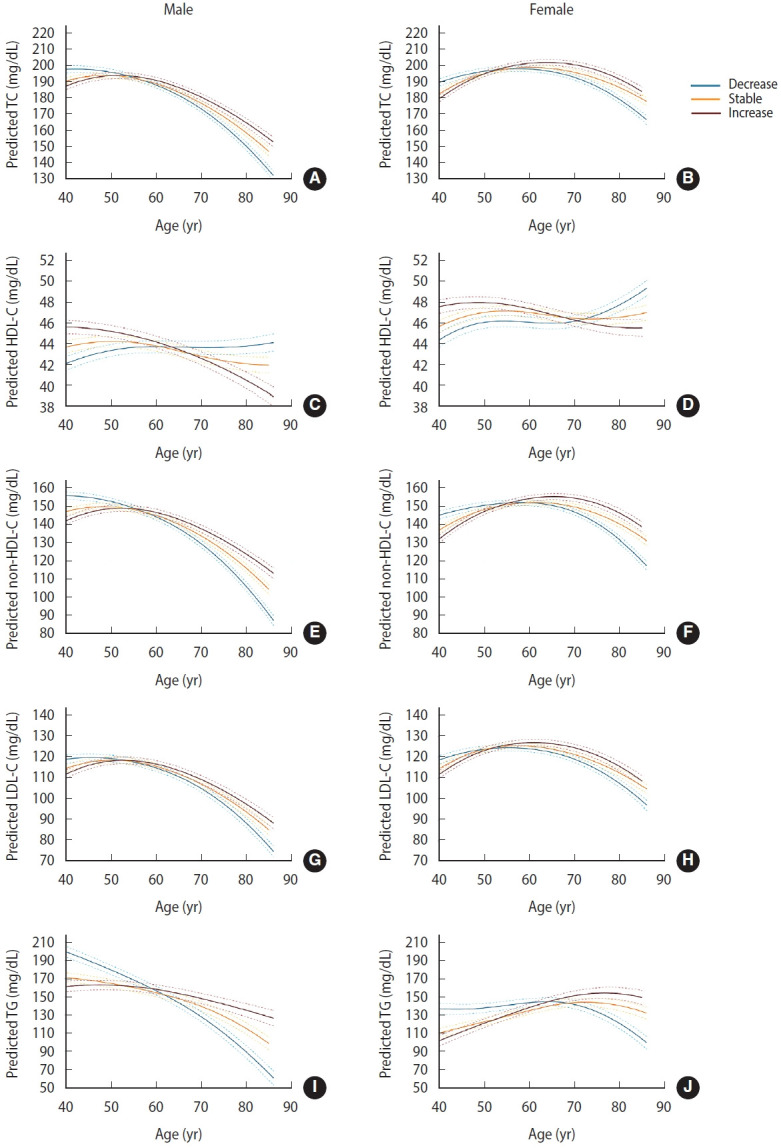
Estimated trajectories of blood lipid and lipoprotein levels with age according to waist circumference change. Predicted mean trajectories of TC are shown in males (A) and females (B), HDL-C in males (C) and females (D), non-HDL-C in males (E) and females (F), LDL-C in males (G) and females (H), and TG in males (I) and females (J). Adjusted for time-varying smoking status, alcohol consumption, leisure-time physical activity, hypertension, diabetes mellitus, cardiovascular disease, lipid-lowering medications, examination date, and waist circumference, as well as baseline area and education level. Predicted trajectories represent covariate-adjusted marginal means estimated from mixed-effects spline models, reflecting an average over the empirical distribution of covariates: continuous covariates were held constant at their sample means, and categorical covariates were weighted by their observed proportions. TC, total cholesterol; HDL-C, high-density lipoprotein cholesterol; LDL-C, low-density lipoprotein cholesterol; TG, triglyceride.

WC tended to increase continuously in both males and females, whereas age-related body mass index remained stable (Supplementary Material 4). WCs from baseline to the final examination among participants with increased WC were 81.8 cm to 90.7 cm in males and 79.3 cm to 88.7 cm in females, respectively. WCs from baseline to the final examination among participants with decreased WC were 85.3 cm to 82.5 cm in males and 86.3 cm to 81.7 cm in females, respectively (Supplementary Materials 5 and 6). According to the mixed model, ΔWC was significantly associated with lipid and lipoprotein levels in both males and females, even after adjusting for time-varying lifestyle factors, diseases, lipid-lowering medication, WC, examination date, and education level at baseline. ΔWC was inversely associated with TC, non-HDL-C, LDL-C, and TG levels and positively associated with HDL-C levels (Table 2). For all 5 lipid and lipoprotein measures, the age–ΔWC interaction was significant (all p<0.05), indicating that the associations between ΔWC and lipid and lipoprotein levels were not consistent across different ages. In the analysis by ΔWC group, males in the decrease group showed the highest levels of TC and LDL-C until the early 50s, non-HDL-C until the mid-50s, and TG until the late 50s, after which these levels declined to the lowest among the groups (Figure 3). In females in the decrease group, the highest values were observed until the early 50s for TC and LDL-C, the mid-50s for non–HDL-C, and the mid-60s for TG, after which they reversed to the lowest values and continued to decline. Both males and females in the decrease group had the lowest HDL-C levels until the 60s in males and the 70s in females, after which HDL-C became the highest among the groups. In males, HDL-C levels in the increase group declined steadily, whereas those in the decrease group increased modestly until age 60 and then plateaued. In females, HDL-C levels in the increase group showed a slight decline after age 50, whereas those in the decrease group increased until age 50, remained stable thereafter, and rose again after the 70s. These trends were similar in males and females with and without abdominal obesity at baseline (Supplementary Materials 7 and 8).

## DISCUSSION

In this prospective cohort of 9,149 Korean adults, we examined trends in lipid and lipoprotein levels with age according to sex and area. The trajectories of lipid and lipoprotein levels exhibited an inverted U-shaped relationship with age. TC, LDL-C, and non-HDL-C levels gradually increased until the late 40s in males and the 60s in females, with subsequent declines thereafter; in contrast, HDL-C levels remained stable in males, whereas in females they increased until the early 50s, declined thereafter, and rose again from the 70s onward. TG levels decreased with advancing age in males, whereas in females they increased up to the age of 70 years and then decreased. TC, non–HDL-C, and LDL-C levels were less favorable in urban than in rural areas. HDL-C levels were significantly higher in urban than in rural areas for both males and females, with the divergence becoming evident after the mid-40s to late 40s in males. Additionally, both males and females with a consistent decrease in WC showed greater improvement in lipid and lipoprotein profiles than those with stable or increased WC.

According to data from the National Health and Nutrition Examination Survey (NHANES), TC, TG, and LDL-C levels decreased from 1999 to 2018 [17], whereas data from the Korea National Health and Nutrition Examination Survey (KNHANES) indicated a slight increase in these markers from 2007 to 2020 [18]. However, the treatment rate for hypercholesterolemia in Korea remained low (55.2%) in 2019-2020, underscoring the need for appropriate management. The Dyslipidemia Fact Sheet 2022, based on the KNHANES, revealed a lower prevalence of dyslipidemia in females than in males among those in their 20s-50s; however, this trend reversed in those aged >60 years [18]. Previous longitudinal studies have shown that TC and LDL-C increase until midlife and then decline thereafter, while HDL-C tends to decrease with advancing age within the same cohorts [7,19]. In contrast, cross-sectional analyses from the NHANES reported that HDL-C levels remained relatively stable across age groups [20]. Our longitudinal prospective study expands on this prior work by examining TC, HDL-C, non–HDL-C, LDL-C, and TG levels. We found greater increases in lipid and lipoprotein levels in females than in males. The gradual decline in LDL-C levels at older ages, with predicted values appearing relatively low, may be partly attributable to the restriction of calculation to participants with TG <400 mg/dL, the smaller sample size in the oldest age groups, and the smoothing characteristics of the mixed-effects model. In our longitudinal analysis, mean LDL-C levels declined and HDL-C increased with advancing age, particularly in older participants. By contrast, the Dyslipidemia Fact Sheet 2024 [21], based on national cross-sectional data, reported that the prevalence of hyper-LDL cholesterolemia and hypo-HDL cholesterolemia increases with age. This discrepancy may reflect differences in outcome metrics (mean concentration vs. prevalence), as well as selective survival, whereby individuals with poorer metabolic health are more likely to die or be lost to follow-up, leaving healthier individuals in the older age groups. In addition, lipid-lowering medication use and nutritional factors may have contributed to the observed patterns. Therefore, the observation of apparently more favorable lipid and lipoprotein levels at older ages should not be interpreted as an intrinsic improvement in lipid metabolism.

Notably, a divergent sex-specific trend in TC and non-HDL-C levels emerged between the late 40s and early 50s, coinciding with significant hormonal changes in females due to the menopausal transition. A KoGES-based study similarly showed marked increases in lipid levels during the menopausal transition and reported that HDL-C levels increased from the premenopausal period until 1-year after menopause, followed by a transient decline and stabilization after 3-year [22]. However, because that study included only females who were premenopausal at baseline, relatively few older participants were represented. In contrast, our study showed a similar pattern, with HDL-C increasing until the mid-50s and then modestly declining; however, we additionally observed a subsequent rise in HDL-C after age 70. Sex differences in lipid and lipoprotein levels are multifactorial and not yet fully elucidated, but several biological mechanisms have been proposed. Estrogen enhances hepatic LDL receptor activity, thereby promoting LDL-C clearance, and upregulates ApoA1 expression and reverse cholesterol transport, contributing to higher HDL-C levels in females [23]. Higher adiponectin concentrations and greater insulin sensitivity in premenopausal females also support more favorable lipid regulation [24]. During the menopausal transition, declining estrogen is associated with increases in TC, LDL-C, and TG, as well as unfavorable alterations in HDL particle size distribution, with a shift toward smaller, less protective particles [25]. After menopause, visceral fat accumulation and reduced insulin sensitivity further contribute to an atherogenic lipid profile [26]. Moreover, males generally accumulate more visceral adiposity, whereas premenopausal females predominantly store subcutaneous fat, which may partially account for sex-related metabolic differences [27,28]. Furthermore, sex-specific gene expression and vascular differences, such as smaller coronary artery diameter in females, may also influence long-term lipid trajectories [29,30].

Despite a general decline in national mortality rates, significant health disparities persist between rural and urban areas [31,32]. Several rural regions experience higher rates of chronic conditions and associated complications [31,33] coupled with limited access to comprehensive healthcare services [34]. Supporting the existence of rural vulnerability, NHANES (1999-2018) data indicated that urban adults diagnosed with diabetes were less likely to have a non-HDL-C level ≥160 mg/dL compared with those in rural areas, especially adults aged ≥65 years [35]. However, our longitudinal analysis revealed a contrasting pattern for atherogenic lipids, with TC, non-HDL-C, and LDL-C levels being consistently higher in urban areas. This tendency is partially consistent with a systematic review and meta-analysis demonstrating disadvantageous TC and LDL-C levels among urban residents [36]. This vulnerability may be attributable to specific lifestyle changes associated with rapid urbanization in Korea, notably increased exposure to Westernized dietary patterns and prolonged sedentary behavior in urban settings. These factors are both established independent risk factors for atherogenic dyslipidemia [37]. This result suggests the necessity for targeted public health interventions focusing on lifestyle modification within urbanized Korean settings. Furthermore, in males, regional disparities in TC, non-HDL-C, and LDL-C levels narrowed with age, reversing after the age of 80 years. A meta-analysis reported no consistent urban–rural differences in HDL-C levels [36]. In contrast, our study found that HDL-C levels were significantly higher in urban than in rural areas for both males and females, with the divergence becoming evident after the mid-40s to late 40s in males. For TG, only females in rural areas displayed higher levels than their urban counterparts. These heterogeneous patterns across lipid fractions suggest that regional disparities are not uniform and likely reflect a complex interplay of healthcare access, lifestyle, and dietary factors. However, because dietary data were not consistently collected in the KoGES, we were unable to directly assess the role of nutrition in these differences.

To our knowledge, this is the first study to investigate trajectories of age-related blood lipid and lipoprotein levels according to long-term ΔWC using biennially repeated measurements. A previous study reported that WC reduction was significantly associated with decreased TC and LDL-C levels but displayed no significant relationship with HDL-C and TG levels [38]. Another study indicated that a greater relative decrease in WC was associated with improvements in TC, HDL-C, LDL-C, and TG levels [39]. Our study found a significant interaction between age and ΔWC. The group exhibiting a decrease in WC had higher initial values, whereas the group with an increase in WC began with lower values. Lipid and lipoprotein levels were the least favorable in the early 40s but improved with advancing age as WC consistently decreased in participants with and without abdominal obesity, suggesting beneficial effects of sustained WC reduction on lipid profiles.

The strengths of this study include its prospective design, biennially standardized lipid and lipoprotein measurements, and repeated assessment of covariates up to 9 times. This study also had several limitations. First, it included relatively healthy, middle-aged participants recruited from 2 specific communities in Korea, which may limit the generalizability of our findings to the broader Korean population. Second, as is common in long-term cohort studies, participants with worsening health were more likely to be lost to follow-up, whereas healthier individuals were more likely to remain in the cohort, potentially influencing the observed results. In our study, participants were stratified into an early termination group (those who discontinued follow-up in 2010 or earlier) and a long-term follow-up group (those who remained under observation beyond 2010). Compared with long-term participants, those in the early termination group had higher prevalences of smoking, hypertension, diabetes, and CVD (Supplementary Materials 9 and 10), suggesting that participants retained for long-term follow-up were relatively healthier. Although we adjusted for major covariates as time-varying variables in the mixed models, this inherent feature of the cohort may still have influenced lipid levels observed in older age groups. Third, we were unable to adjust for important covariates that may influence lipid metabolism, such as dietary intake, menopausal status, and hormonal therapy, because these variables were not consistently collected across all survey waves in the KoGES. Although major lifestyle and clinical factors were accounted for through time-varying adjustments, the absence of these additional covariates may have influenced the results. Finally, the wide age range at baseline, spanning 40-69 years, may have yielded cohort effects owing to variations in birth cohorts. A previous study reported that younger generations had more favorable lipid profiles [8]. Based on this evidence, we also examined lipid profiles by baseline age group in the present study (Supplementary Material 11). Participants aged 60-69 years at baseline had lower HDL-C levels compared with younger baseline age groups in both males and females. In contrast, females in the older baseline age group exhibited higher TC, non–HDL-C, LDL-C, and TG levels. Among males, the older baseline age group showed slightly higher TG levels, whereas TC, non–HDL-C, and LDL-C displayed comparable patterns across baseline age groups. Considering the potential variations in lipid and lipoprotein trends across generations owing to lifestyle and dietary differences, we analyzed a mixed model adjusted for examination date.

Although our study characterized age-related trajectories of lipid and lipoprotein levels by sex, region, and ΔWC using longitudinal data, further research is warranted to clarify how these changes translate into absolute cardiovascular risk and long-term clinical outcomes.

In summary, we analyzed lipid and lipoprotein level trajectories from midlife to late life in a sex- stratified and area-stratified manner and observed a significant interaction between age and sex in each trajectory, with trends suggesting that lipid and lipoprotein levels in females appear to become similar to those in males by midlife. In particular, in sex-stratified analyses of longitudinal data with serial lipid and lipoprotein levels compared with baseline levels, females showed a steeper increase in all 5 lipid and lipoprotein measures than males. Independent of time-varying covariates, a decrease in WC significantly improved lipid and lipoprotein profiles. Therefore, effective management of abdominal obesity is crucial for maintaining favorable lipid and lipoprotein levels, emphasizing the need for tailored approaches based on age and sex for optimal abdominal care.

## Figures and Tables

**Figure f4-epih-47-e2025066:**
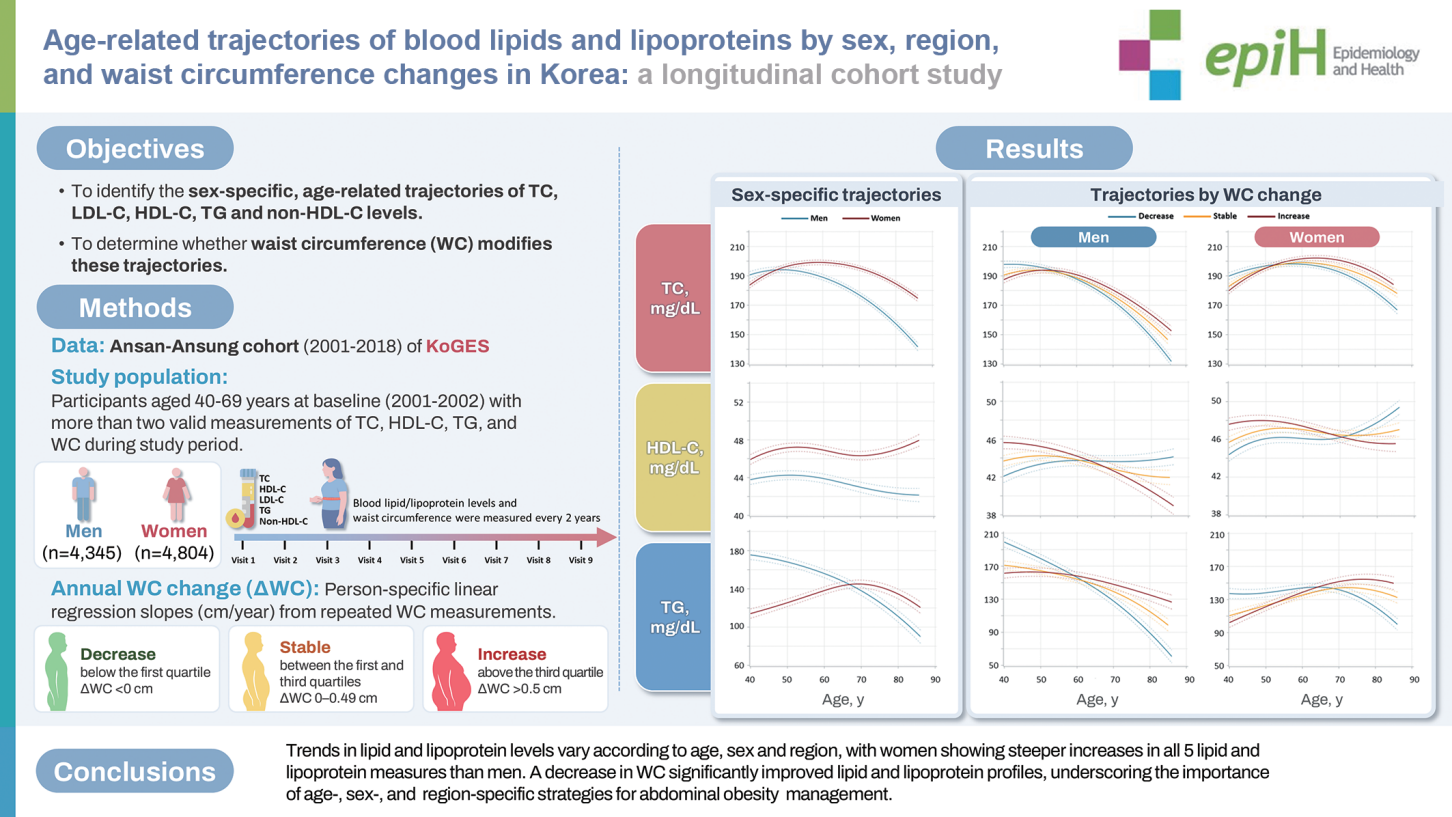


**Table 1.** Characteristics of study participants at the first and last attended examinations

Characteristics	Baseline examination			Final examination		
	Male (n=4,345)	Female (n=4,804)	p-value	Male (n=4,345)	Female (n=4,804)	p-value
Age (yr)	51.9±8.8	52.7±9.0	<0.001	64.8±9.4	66.0±9.6	<0.001
Lifestyle variables						
Current smoker	2,212 (49.1)	172 (3.7)	<0.001	1,126 (26.0)	82 (1.7)	<0.001
Current drinker	3,080 (71.3)	1,217 (25.7)	<0.001	2,626 (60.6)	773 (16.1)	<0.001
Leisure time physical inactivity	3,022 (69.6)	3,594 (74.8)	<0.001	2,235 (51.4)	2,636 (54.9)	0.001
Clinical characteristics						
Body mass index (kg/m^2^)	24.2±2.9	24.9±3.3	<0.001	24.2±3.1	24.8±3.5	<0.001
Waist circumference (cm)	83.7±7.7	82.0±9.7	<0.001	86.6±8.3	84.7±9.6	<0.001
Systolic blood pressure (mmHg)	122.0±16.9	121.2±19.5	0.045	123.6±16.4	123.9±18.4	0.479
Diastolic blood pressure (mmHg)	81.8±10.9	79.0±11.7	<0.001	78.4±10.1	76.3±9.8	<0.001
Hypertension	1,370 (31.5)	1,457 (30.3)	0.214	2,106 (48.5)	2,447 (51.0)	0.018
Diabetes mellitus	377 (8.7)	363 (7.6)	0.045	876 (20.9)	934 (19.8)	0.209
History of CVD	148 (3.4)	127 (2.6)	0.033	356 (8.2)	318 (6.6)	0.004
Antihypertensive treatment	371 (8.6)	626 (13.1)	<0.001	1,523 (36.2)	1,978 (41.9)	<0.001
Lipid-lowering treatment	21 (0.5)	17 (0.4)	0.336	482 (11.1)	890 (18.5)	<0.001
Laboratory examinations (mg/dL)						
Total cholesterol	191.1±35.8	190.8±35.0	0.748	180.7±37.2	193.1±37.9	<0.001
HDL-C	43.5±10.0	45.6±10.0	<0.001	43.4±11.6	47.8±11.8	<0.001
LDL-C	114.0±33.0	116.3±30.8	<0.001	109.4±32.4	119.0±33.6	<0.001
TG	178.0±119.1	148.3±87.0	<0.001	147.5±110.2	133.8±75.4	<0.001
Non-HDL-C	147.5±35.5	145.3±34.0	0.002	137.3±35.7	145.4±35.8	<0.001
Fasting plasma glucose	89.6±22.7	84.8±18.4	<0.001	101.9±27.8	97.6±26.3	<0.001

Values are presented as means±standard deviations or number (%); The final examination date differed among participants. CVD, cardiovascular disease; HDL-C, high-density lipoprotein cholesterol; LDL-C, low-density lipoprotein cholesterol; TG, triglyceride.

**Table 2.** Population-averaged estimates for age and WC change for lipid and lipoprotein levels from mixed models in male and female participants^1^

Variables	TC	HDL-C	Non-HDL-C	LDL-C	TG
Overall					
Age	5.231 (0.139)^***^	0.367 (0.037)^***^	4.587 (0.131)^***^	4.186 (0.127)^***^	3.136 (0.289)^***^
Age^2^	-0.040 (0.001)^***^	-0.002 (0.000)^***^	-0.035 (0.001)^***^	-0.032 (0.001)^***^	-0.023 (0.002)^***^
Sex (male)	41.353 (2.177)^***^	4.068 (0.606)^***^	34.483 (2.066)^***^	19.582 (1.969)^***^	84.318 (4.498)^***^
Area (rural)	-9.633 (0.650)^***^	0.454 (0.209)^*^	-12.417 (0.626)^***^	-8.806 (0.567)^***^	-10.677 (1.315)
Age×Sex	-0.912 (0.035)^***^	-0.124 (0.010)^***^	-0.760 (0.033)^***^	-0.510 (0.032)^***^	-1.346 (0.072)^***^
Male					
Age	0.747 (0.197)^***^	0.185 (0.054)^***^	0.575 (0.188)^**^	0.604 (0.181)^***^	-0.140 (0.639)
Age^2^	-0.008 (0.002)^***^	-0.001 (0.000)^*^	-0.007 (0.002)^***^	-0.006 (0.001)^***^	-0.011 (0.005)^*^
Area (rural)	-11.648 (0.950)^***^	0.791 (0.297)^**^	-12.464 (0.922)^***^	-11.531 (0.844)^***^	-2.741 (2.870)
WC change	-8.094 (2.671)^**^	2.903 (0.790)^***^	-10.637 (2.574)^***^	-5.312 (2.415)^*^	-26.918 (8.314)^**^
Age×WC change	0.142 (0.045)^**^	-0.047 (0.014)^***^	0.182 (0.044)^***^	0.093 (0.041)^**^	0.438 (0.143)^**^
Female					
Age	7.769 (0.190)^***^	0.302 (0.051)^***^	7.238 (0.180)^***^	6.372 (0.173)^***^	6.082 (0.442)^***^
Age^2^	-0.062 (0.002)^***^	-0.003 (0.000)^***^	-0.058 (0.001)^***^	-0.051 (0.001)^***^	-0.046 (0.004)^***^
Area (rural)	-10.180 (0.883)^***^	-1.391 (0.294)^***^	-11.310 (0.866)^***^	-8.121 (0.758)^***^	-3.942 (2.019)
WC change	-6.351 (2.224)^**^	6.010 (0.685)^***^	-5.061 (2.173)^*^	-5.804 (1.958)^**^	-23.635 (5.122)^***^
Age×WC change	0.115 (0.038)^**^	-0.102 (0.011)^***^	0.086 (0.037)^*^	0.103 (0.034)^**^	0.417 (0.088)^***^

Values are presented as regression coefficient (standard error). WC, waist circumference; TC, total cholesterol; HDL-C, high-density lipoprotein cholesterol; LDL-C, low-density lipoprotein cholesterol; TG, triglyceride.

1 Adjusted for time-varying WC, smoking status, alcohol consumption, leisure time physical activity, hypertension, diabetes mellitus, cardiovascular disease, lipid-lowering medication, and examination date, and baseline area and education level.

* p<0.05,

** p<0.01,

*** p<0.001.

## Supplementary materials

Supplementary Material 1. Number of visits and follow-ups according to age group at baseline and waist circumference (WC) trajectory groups

Supplementary Material 2. Frequency of participants according to year at last visit

Supplementary Material 3. The estimated sex-specific trajectories of blood lipid and lipoprotein levels with aging in subjects without lipid-lowering medications

Supplementary Material 4. The estimated sex-specific trajectories of waist circumference and body mass index with aging

Supplementary Material 5. Characteristics of the study population at the first and last attended examinations according to waist circumference trajectory groups in males

Supplementary Material 6. Characteristics of the study population at the first and last attended examinations according to waist circumference trajectory groups in females

Supplementary Material 7. The estimated trajectories of blood lipid with aging by waist circumference change in male with and without abdominal obesity Adjusted for time-varying smoking status, alcohol consumption, leisure-time physical activity, hypertension, diabetes mellitus, cardiovascular disease, lipid-lowering medication, waist circumference, and examination date, as well as baseline area and education level

Supplementary Material 8. The estimated trajectories of blood lipid with aging by waist circumference change in female with and without abdominal obesity Adjusted for time-varying smoking status, alcohol consumption, leisure-time physical activity, hypertension, diabetes mellitus, cardiovascular disease, lipid-lowering medication, waist circumference, and examination date, as well as baseline area and education level

Supplementary Material 9. Baseline characteristics according to follow-up duration in males

Supplementary Material 10. Baseline characteristics according to follow-up duration in females

Supplementary Material 11. The estimated trajectories of blood lipid with aging by age group at baseline Adjusted for time-varying smoking status, alcohol consumption, leisure-time physical activity, hypertension, diabetes mellitus, cardiovascular disease, lipid-lowering medication, waist circumference, and examination date, as well as baseline area and education level
