# Supplementary material for: Age-related trajectories of blood lipids and lipoproteins by sex, region, and waist circumference changes in Korea: a longitudinal cohort study
Source: Epidemiol Health. 2025 Dec 9;47:e2025066. doi: 10.4178/epih.e2025066 (PMC12884011; doi:10.4178/epih.e2025066)
Supplement: Supplementary Material 1. — Number of visits and follow-ups according to age group at baseline and waist circumference (WC) trajectory groups [file epih-47-e2025066-Supplementary-1.pdf]

Supplementary Material 1. Number of visits and follow-ups according to age group at baseline and waist circumference (WC) trajectory groups

	**Number of visits**	**Follow-up time**, yrs
**Overall (n = 9,149)**	7.7 ± 2.3	13.1 ± 4.6
**Age groups at baseline**, yrs		
40–49 (n = 4,288)	7.9 ± 2.2	13.5 ± 4.3
50–59 (n = 2,410)	7.8 ± 2.2	13.5 ± 4.3
60–69 (n = 2,451)	7.1 ± 2.6	12.0 ± 5.1
**WC trajectory groups**		
Decrease (n = 713)	5.2 ± 2.9	8.3 ± 5.8
Stable (n = 5,978)	8.2 ± 1.7	14.3 ± 3.3
Increase (n = 2,458)	7.0 ± 2.7	11.7 ± 5.4

Data are reported as means ± standard deviations.
