# Supplementary material for: Age-related trajectories of blood lipids and lipoproteins by sex, region, and waist circumference changes in Korea: a longitudinal cohort study
Source: Epidemiol Health. 2025 Dec 9;47:e2025066. doi: 10.4178/epih.e2025066 (PMC12884011; doi:10.4178/epih.e2025066)
Supplement: Supplementary Material 2. — Frequency of participants according to year at last visit [file epih-47-e2025066-Supplementary-2.pdf]

Supplementary Material 2. Frequency of participants according to year at last visit

Year at last visit	No. of participants (%)
**2003-2004**	624 (6.8)
**2005-2006**	455 (5.0)
**2007-2008**	292 (3.2)
**2009-2010**	360 (3.9)
**2011-2012**	362 (4.0)
**2013-2014**	298 (3.3)
**2015-2016**	601 (6.6)
**2017-2018**	615.3 (67.3)
