# Supplementary material for: Age-related trajectories of blood lipids and lipoproteins by sex, region, and waist circumference changes in Korea: a longitudinal cohort study
Source: Epidemiol Health. 2025 Dec 9;47:e2025066. doi: 10.4178/epih.e2025066 (PMC12884011; doi:10.4178/epih.e2025066)
Supplement: Supplementary Material 3. — The estimated sex-specific trajectories of blood lipid and lipoprotein levels with aging in subjects without lipid-lowering medications [file epih-47-e2025066-Supplementary-3.pdf]

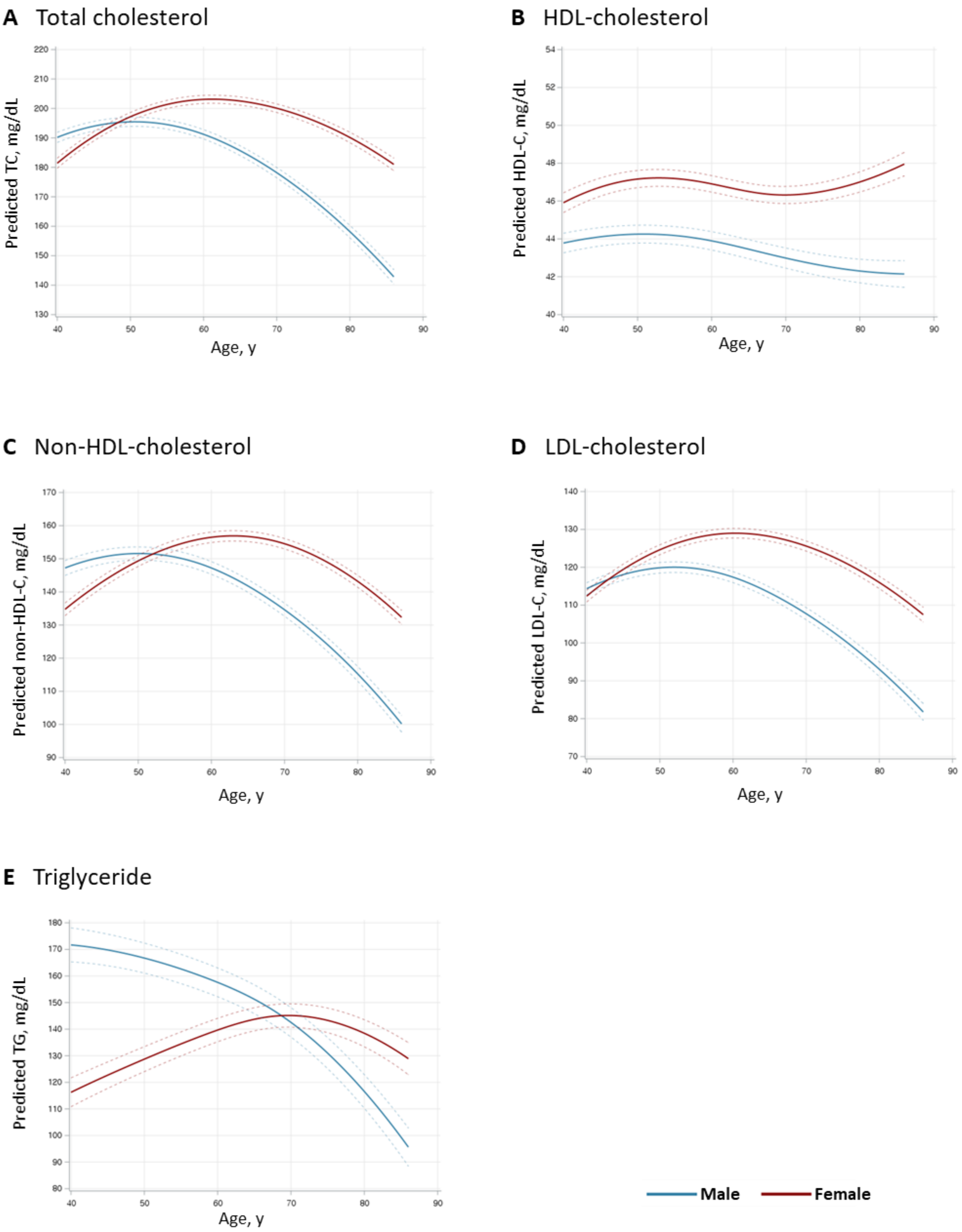

Supplementary Material 3. The estimated sex-specific trajectories of blood lipid and lipoprotein levels with aging in subjects without lipid-lowering medications
