# Supplementary material for: Age-related trajectories of blood lipids and lipoproteins by sex, region, and waist circumference changes in Korea: a longitudinal cohort study
Source: Epidemiol Health. 2025 Dec 9;47:e2025066. doi: 10.4178/epih.e2025066 (PMC12884011; doi:10.4178/epih.e2025066)
Supplement: Supplementary Material 5. — Characteristics of the study population at the first and last attended examinations according to waist circumference trajectory groups in males [file epih-47-e2025066-Supplementary-5.pdf]

Supplementary Material 5. Characteristics of the study population at the first and last attended examinations according to waist circumference trajectory groups in males

Variables	Decrease (ΔWC < 0 cm) (n=1,239)		Stable (ΔWC 0 – 0.5 cm) (n=1,972)		Increase (ΔWC > 0.5 cm) (n=1,134)	
	Baseline examination	Final examination	Baseline examination	Final examination	Baseline examination	Final examination
**Age**, yrs	52.6±8.82	64.6±9.85	51.1±8.35	65.4±8.74	52.6±9.32	64.0±9.89
**Lifestyle variables**						
**Current smoker**	557 (45.1)	363 (29.3)	909 (46.3)	458 (23.3)	656 (58.4)	305 (27.0)
**Current drinker**	888 (71.9)	735 (59.4)	1394 (71.1)	1196 (60.7)	798 (71.0)	695 (61.6)
**Leisure time physical inactivity**	839 (67.7)	575 (46.4)	1339 (67.9)	1000 (50.7)	844 (74.4)	660 (58.2)
**Clinical characteristics**						
**BMI**, kg/m^2	24.7±2.91	23.1±2.96	24.3±2.87	24.3±2.93	23.6±2.94	25.0±3.2
**Waist circumference**, cm	85.8±7.5	82.5±7.54	83.5±7.43	86.9±7.45	81.8±7.67	90.7±8.33
**Systolic blood pressure**, mmHg	123.3±17.02	122.0±16.9	121.4±16.63	124.0±15.72	121.6±17.34	124.7±16.93
**Diastolic blood pressure**, mmHg	82.5±10.79	77.0±10.51	81.8±10.82	78.6±9.68	81.1±10.98	79.7±10.05
**Hypertension**	432 (34.9)	530 (42.8)	602 (30.5)	1000 (50.7)	336 (29.6)	576 (50.8)
**Diabetes mellitus**	157 (12.8)	303 (25.2)	139 (7.1)	394 (20.8)	81 (7.2)	179 (16.2)
**History of CVD**	51 (4.1)	94 (7.6)	57 (2.9)	169 (8.6)	40 (3.5)	93 (8.2)
**Antihypertensive treatment**	119 (9.7)	384 (32.0)	165 (8.4)	745 (39.2)	87 (7.7)	394 (35.8)
**Lipid-lowering treatment**	8 (0.7)	128 (10.3)	9 (0.5)	250 (12.7)	4 (0.4)	104 (9.2)
**Laboratory examinations**						
**Total cholesterol**, mg/dL	194.0±37.21	177.6±38.15	190.9±34.52	180.6±36.26	188.1±36.26	184.2±37.3
**HDL-C**, mg/dL	42.3±9.79	44.4±12.26	43.5±9.63	43.5±11.37	44.9±10.53	42.0±10.95
**LDL-C**, mg/dL	115.2±33.9	107.4±32.92	114.4±31.84	109.3±32.15	111.9±33.66	112.1±32.28
**TG**, mg/dL	197.8±143.15	138.3±125.68	172.5±104.29	146.2±101.87	165.8±111.38	159.7±104.96
**Non-HDL-C**, mg/dL	151.7±36.93	133.2±36.49	147.4±34.11	137.0±34.72	143.1±35.65	142.2±35.77
**Fasting plasma glucose**, mg/dL	92.8±25.4	104.7±34.42	88.7±21.31	101.0±24.39	87.9±21.75	100.4±24.85

Continuous variables are reported as means ± standard deviations, and categorical variables are reported as n (%).

Abbreviations: HDL-C, high-density lipoprotein cholesterol; LDL-C, low-density lipoprotein cholesterol; TG, triglyceride; non-HDL-C, non-high-density lipoprotein cholesterol; BMI, body mass index; CVD, cardiovascular disease; WC, waist circumference

The final examination date for each participant was different.
