# Supplementary material for: Age-related trajectories of blood lipids and lipoproteins by sex, region, and waist circumference changes in Korea: a longitudinal cohort study
Source: Epidemiol Health. 2025 Dec 9;47:e2025066. doi: 10.4178/epih.e2025066 (PMC12884011; doi:10.4178/epih.e2025066)
Supplement: Supplementary Material 6. — Characteristics of the study population at the first and last attended examinations according to waist circumference trajectory groups in females [file epih-47-e2025066-Supplementary-6.pdf]

Supplementary Material 6. Characteristics of the study population at the first and last attended examinations according to waist circumference trajectory groups in females

Variables	Decrease (ΔWC < 0 cm) (n=1,239)		Stable (ΔWC 0 – 0.5 cm) (n=1,972)		Increase (ΔWC > 0.5 cm) (n=1,134)	
	**Baseline examination**	**Final examination**	**Baseline examination**	**Final examination**	**Baseline examination**	**Final examination**
**Age**, yrs	55.4±9.27	68.0±10.53	51.7±8.5	66.3±8.72	51.5±8.92	63.4±9.2
**Lifestyle variables**						
**Current smoker**	46 (3.3)	26 (1.8)	58 (2.9)	26 (1.3)	68 (5.3)	30 (2.3)
**Current drinker**	304 (21.8)	184 (13.0)	558 (27.3)	330 (16.0)	355 (27.2)	259 (19.6)
**Leisure time physical inactivity**	1090 (77.0)	810 (57.2)	1515 (73.4)	1065 (51.6)	989 (74.7)	761 (57.5)
**Clinical characteristics**						
**BMI**, kg/m^2	25.4±3.39	23.8±3.33	24.7±3.13	24.6±3.15	24.7±3.37	26.0±3.64
**Waist circumference**, cm	86.3±9.77	81.7±9.48	80.7±9.02	84.2±8.88	79.3±9.00	88.7±9.56
**Systolic blood pressure**, mmHg	125.3±19.68	125.2±19.55	119.6±18.82	123.7±18.11	119.4±19.68	122.8±17.40
**Diastolic blood pressure**, mmHg	81.0±11.78	75.5±10.11	78.2±11.34	76.2±9.44	78.1±11.75	77.4±9.84
**Hypertension**	563 (39.8)	789 (55.7)	542 (26.3)	1030 (49.9)	352 (26.6)	628 (47.4)
**Diabetes mellitus**	183 (13.0)	381 (27.5)	112 (5.4)	359 (17.7)	68 (5.1)	194 (14.8)
**History of CVD**	44 (3.1)	95 (6.7)	49 (2.4)	148 (7.2)	34 (2.6)	75 (5.7)
**Antihypertensive treatment**	256 (18.2)	647 (46.8)	225 (10.9)	836 (41.3)	145 (11.0)	495 (37.7)
**Lipid-lowering treatment**	6 (0.4)	254 (17.9)	7 (0.3)	426 (20.6)	4 (0.3)	210 (15.9)
**Laboratory examinations**						
**Total cholesterol**, mg/dL	194.1±34.36	187.8±38.35	189.3±34.34	193.0±37.41	189.6±36.56	199.0±37.44
**HDL-C**, mg/dL	44.1±9.59	48.0±12.42	45.7±9.77	47.9±11.58	47.0±10.52	47.4±11.45
**LDL-C**, mg/dL	118.5±30.25	114.5±33.56	115.3±30.81	118.7±33.16	115.5±31.19	124.1±33.59
**TG**, mg/dL	162.1±88.52	128.9±77.38	144.7±83.96	132.7±71.8	139.2±88.39	140.9±78.01
**Non-HDL-C**, mg/dL	150.0±33.1	139.8±35.58	143.7±33.66	145.1±35.52	142.7±34.95	151.6±35.61
**Fasting plasma glucose**, mg/dL	87.1±20.43	100.4±31.55	84.2±17.96	96.4±23.12	83.2±16.42	96.5±24.66

Continuous variables are reported as means ± standard deviations, and categorical variables are reported as n (%).

Abbreviations: HDL-C, high-density lipoprotein cholesterol; LDL-C, low-density lipoprotein cholesterol; TG, triglyceride; non-HDL-C, non-high-density lipoprotein cholesterol; BMI, body mass index; CVD, cardiovascular disease

The final examination date for each participant was different.
