# Supplementary material for: Age-related trajectories of blood lipids and lipoproteins by sex, region, and waist circumference changes in Korea: a longitudinal cohort study
Source: Epidemiol Health. 2025 Dec 9;47:e2025066. doi: 10.4178/epih.e2025066 (PMC12884011; doi:10.4178/epih.e2025066)
Supplement: Supplementary Material 7. — The estimated trajectories of blood lipid with aging by waist circumference change in male with and without abdominal obesity Adjusted for time-varying smoking status, alcohol consumption, leisure-time physical activity, hypertension, diabetes mellitus, cardiovascular disease, lipid-lowering medication, waist circumference, and examination date, as well as baseline area and education level [file epih-47-e2025066-Supplementary-7.pdf]

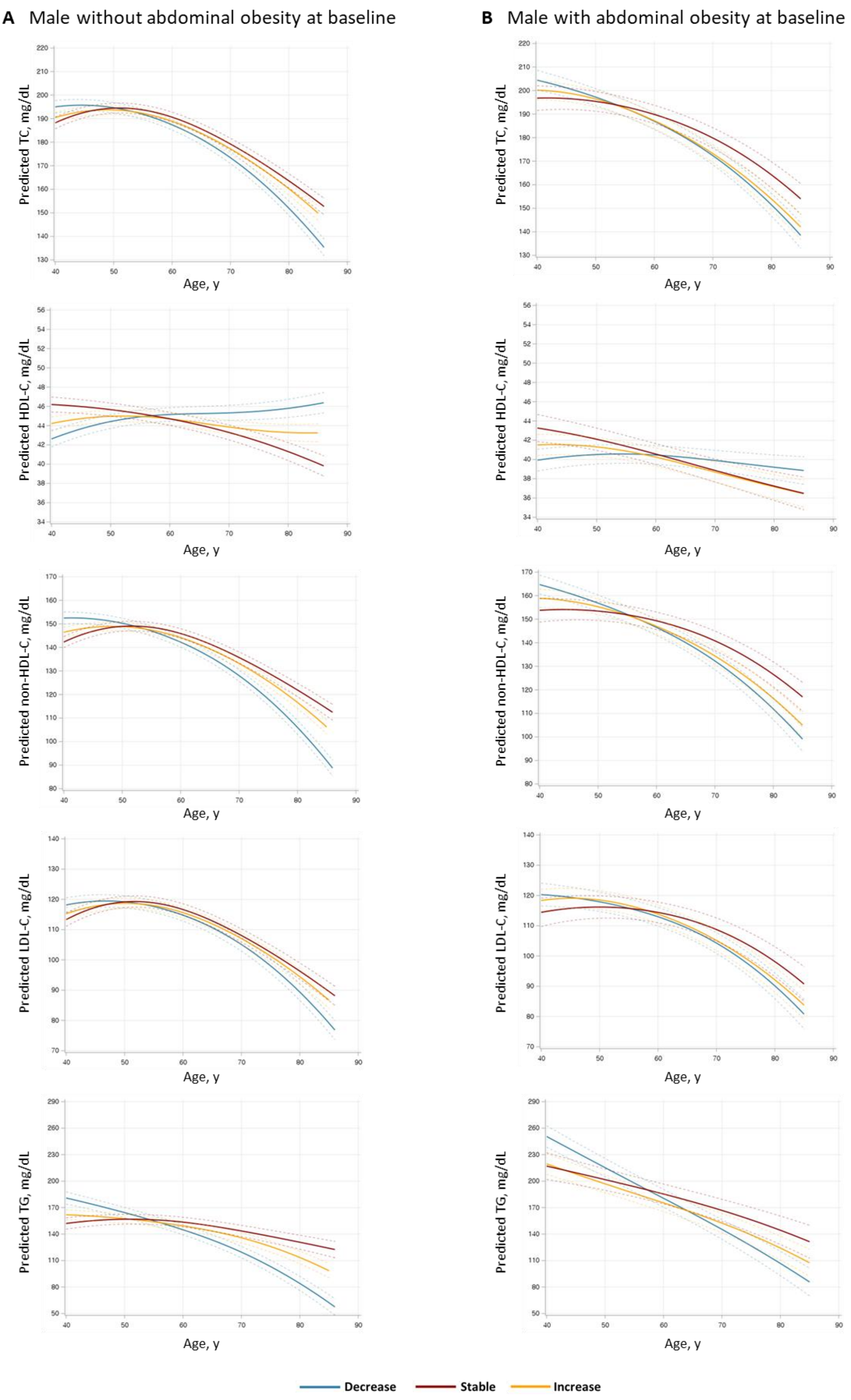

Supplementary Material 7. The estimated trajectories of blood lipid with aging by waist circumference change in male with and without abdominal obesity

Adjusted for time-varying smoking status, alcohol consumption, leisure-time physical activity, hypertension, diabetes mellitus, cardiovascular disease, lipid-lowering medication, waist circumference, and examination date, as well as baseline area and education level
