# Supplementary material for: Age-related trajectories of blood lipids and lipoproteins by sex, region, and waist circumference changes in Korea: a longitudinal cohort study
Source: Epidemiol Health. 2025 Dec 9;47:e2025066. doi: 10.4178/epih.e2025066 (PMC12884011; doi:10.4178/epih.e2025066)
Supplement: Supplementary Material 9. — Baseline characteristics according to follow-up duration in males [file epih-47-e2025066-Supplementary-9.pdf]

Supplementary Material 9. Baseline characteristics according to follow-up duration in males

Characteristics	Early termination (≤2010) (n=891)	Long-term follow-up (>2010) (n=3432)	P-value
**Age**, yrs	53.8±9.8	51.4±8.4	<0.001
**Lifestyle variables**			
**Current smoker**	491 (55.1)	1631 (47.5)	<0.001
**Current drinker**	628 (70.3)	2452 (71.5)	0.720
**Leisure time physical inactivity**	646 (72.5)	2354 (68.6)	0.025
**Clinical characteristics**			
**BMI**, kg/m^2	23.9±3.0	24.3±2.9	<0.001
**Waist circumference**, cm	83.4±7.9	83.8±7.6	0.134
**Systolic blood pressure**, mmHg	124.2±18.8	121.4±16.4	<0.001
**Diastolic blood pressure**, mmHg	82.4±11.3	81.7±10.7	0.096
**Hypertension**	321 (35.9)	1049 (30.4)	0.002
**Diabetes mellitus**	107 (12.1)	270 (7.9)	<0.001
**History of CVD**	56 (6.3)	92 (2.7)	<0.001
**Antihypertensive treatment**	98 (11.1)	273 (8.0)	0.003
**Lipid-lowering treatment**	5 (0.6)	16 (0.5)	0.715
**Laboratory examinations**			
**Total cholesterol**, mg/dL	187.9±37.8	191.9±35.2	0.003
**HDL-C**, mg/dL	44.4±11.0	43.3±9.7	0.005
**LDL-C**, mg/dL	110.4±34.9	114.9±32.3	<0.001
**TG**, mg/dL	178.7±125.0	177.8±117.5	0.832
**Non-HDL-C**, mg/dL	143.5±38.1	148.6±34.7	<0.001
**Fasting plasma glucose**, mg/dL	91.4±27.0	89.2±21.5	0.009

Continuous variables are reported as means ± standard deviations, and categorical variables are reported as n (%).

Abbreviations: HDL-C, high-density lipoprotein cholesterol; LDL-C, low-density lipoprotein cholesterol; TG, triglyceride; non-HDL-C, non-high-density lipoprotein cholesterol; BMI, body mass index; CVD, cardiovascular disease

The final examination date for each participant was different.
