# Supplementary material for: Age-related trajectories of blood lipids and lipoproteins by sex, region, and waist circumference changes in Korea: a longitudinal cohort study
Source: Epidemiol Health. 2025 Dec 9;47:e2025066. doi: 10.4178/epih.e2025066 (PMC12884011; doi:10.4178/epih.e2025066)
Supplement: Supplementary Material 10. — Baseline characteristics according to follow-up duration in females [file epih-47-e2025066-Supplementary-10.pdf]

Supplementary Material 10. Baseline characteristics according to follow-up duration in females

Characteristics	Early termination (≤2010) (n=836)	Long-term follow-up (>2010) (n=3968)	P-value
**Age**, yrs	54.7±9.9	52.3±8.8	<0.001
**Lifestyle variables**			
**Current smoker**	50 (6.1)	122 (3.1)	<0.001
**Current drinker**	196 (23.8)	1021 (26.0)	0.248
**Leisure time physical inactivity**	632 (75.6)	2961 (74.6)	0.507
**Clinical characteristics**			
**BMI**, kg/m^2	25.0±3.6	24.9±3.2	0.284
**Waist circumference**, cm	82.8±10.4	81.8±9.5	0.005
**Systolic blood pressure**, mmHg	123.7±21.4	120.7±19.0	<0.001
**Diastolic blood pressure**, mmHg	79.7±12.5	78.9±11.5	0.051
**Hypertension**	313 (37.4)	1144 (28.8)	<0.001
**Diabetes mellitus**	89 (10.8)	274 (6.9)	<0.001
**History of CVD**	32 (3.8)	95 (2.4)	0.019
**Antihypertensive treatment**	147 (17.8)	479 (12.1)	<0.001
**Lipid-lowering treatment**	3 (0.4)	14 (0.4)	1.000
**Laboratory examinations**			
**Total cholesterol**, mg/dL	195.8±37.4	189.8±34.4	<0.001
**HDL-C**, mg/dL	45.7±10.3	45.5±9.9	0.653
**LDL-C**, mg/dL	120.1±32.4	115.5±30.4	<0.001
**TG**, mg/dL	153.0±96.5	147.3±84.9	0.089
**Non-HDL-C**, mg/dL	150.1±36.2	144.2±33.4	<0.001
**Fasting plasma glucose**, mg/dL	86.8±21.7	84.4±17.6	0.003

Continuous variables are reported as means ± standard deviations, and categorical variables are reported as n (%).

Abbreviations: HDL-C, high-density lipoprotein cholesterol; LDL-C, low-density lipoprotein cholesterol; TG, triglyceride; non-HDL-C, non-high-density lipoprotein cholesterol; BMI, body mass index; CVD, cardiovascular disease

The final examination date for each participant was different.
